# Viscosynechiolysis and Aspiration-Based Management in Uveitic Cataract Surgery: A Case Report

**DOI:** 10.7759/cureus.105790

**Published:** 2026-03-24

**Authors:** Muralidhar Parri, Kiratmeet Singh

**Affiliations:** 1 Ophthalmology, All India Institute of Medical Sciences, Mangalagiri, Mangalagiri, IND

**Keywords:** breadcrumb appearance, complicated cataract, posterior chamber intraocular lens, posterior synechiae, simcoe aspiration

## Abstract

A 31-year-old female patient with a history of chronic uveitis presented with left eye decreased vision for the last two months. Examination revealed a left eye complicated cataract with dense posterior synechiae and poor pupillary dilation. Cataract is a common complication associated with chronic intraocular inflammation that develops with each uveitic episode. Surgical management of such a cataract is complicated by posterior synechiae, poor pupillary dilation, zonular weakness, and altered lens consistency. Conventional techniques may increase stress on intraocular structures and induce high postoperative inflammation. Viscosynechiolysis provides safe, controlled synechiae release with minimal iris trauma. In this case, recognition of the breadcrumb-like nucleus consistency allowed aspiration-based management, avoiding phacoemulsification energy and thereby reducing zonular stress. The intraoperative course was uneventful, and the patient had a stable postoperative recovery with a favourable visual outcome. This case highlights the importance of recognizing altered nuclear consistency in complicated uveitic cataracts and demonstrates that aspiration-based removal after viscosynechiolysis can be a safe and effective surgical strategy.

## Introduction

Cataractogenesis in uveitic eyes is accelerated due to recurrent inflammation [1]. The incidence of complicated cataract in eyes with prior uveitis has been reported as five to six per 100 eye-years [2]. Unlike age-related cataracts, uveitic cataracts are frequently associated with sequelae such as anterior chamber membranes, vitreous opacities, cystoid macular edema, and secondary glaucoma.

The lens in a complicated cataract may often demonstrate a friable, breadcrumb-like consistency, compounded by posterior synechiae, iris atrophy, pupillary membranes, and angle neovascularization. Optimal management requires strict preoperative control of inflammation, typically with a minimum three-month quiescent interval, perioperative systemic and topical corticosteroids, and cycloplegic-mydriatic agents. Intraoperatively, pupil management is critical. This report emphasizes viscosynechiolysis as a safe, reproducible technique for synechiae release and describes aspiration-based management tailored to the unique lens consistency.

## Case presentation

**Figure 1:**
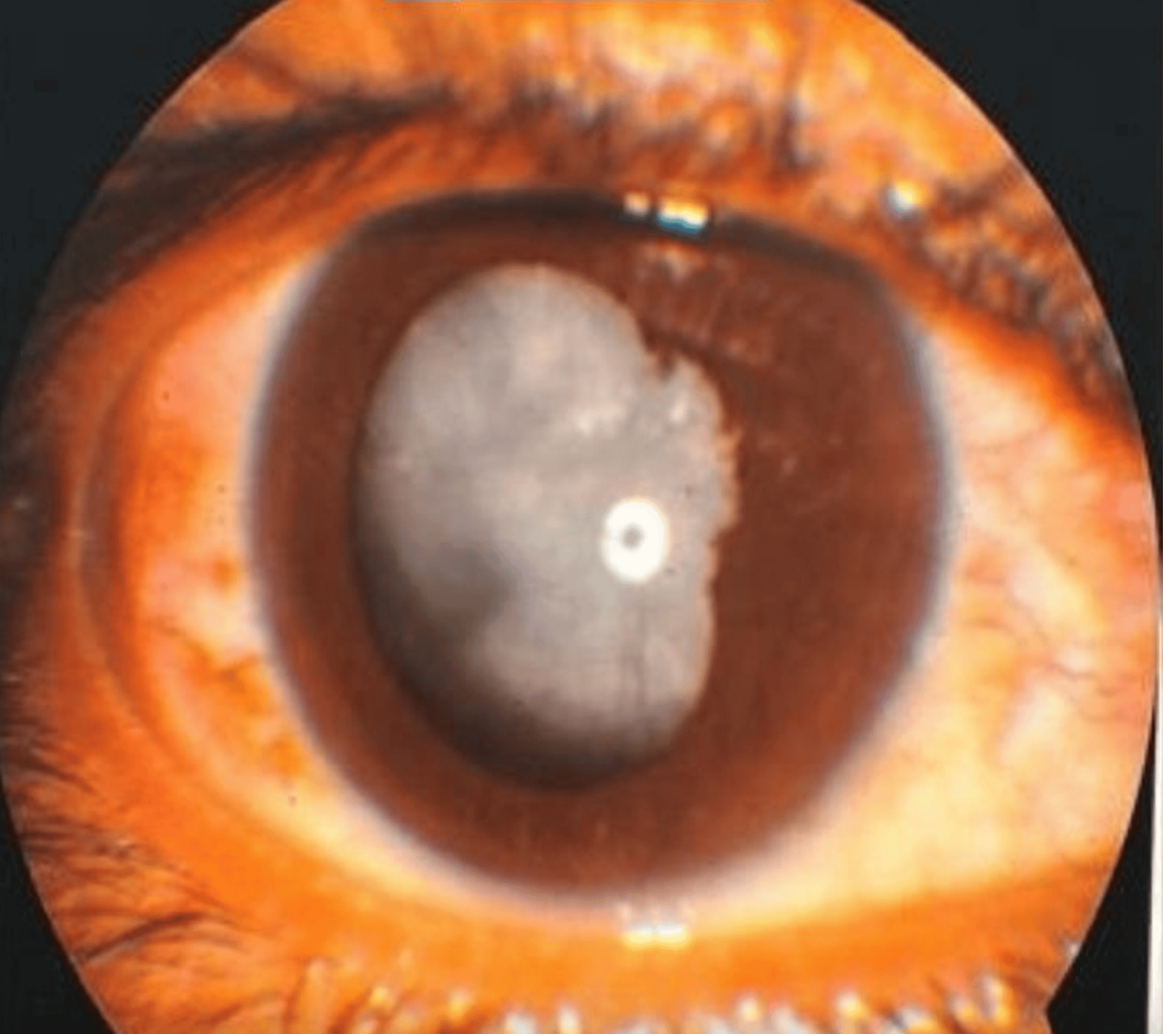
Preoperative picture of complicated uveitic cataract

A 31-year-old female patient presented with a history of diminution of vision in the left eye for the last two months. In the past, the patient had frequent episodes of redness in the right eye and was a known case of left eye non-granulomatous anterior uveitis, which was currently in remission. On examination, the best-corrected visual acuity (BCVA) was 6/9 in the right eye and 6/18 in the left eye. On slit lamp examination, the right eye anterior segment and fundus were within normal limits. The left eye anterior segment examination revealed a few keratic precipitates on the corneal endothelium; the anterior chamber was quiet, with dense posterior synechiae more at 8-9 o'clock positions. There was a complicated cataract. B-scan ultrasonography was unremarkable. The preoperative picture is shown in Figure 1.

After complete preoperative investigations and confirmation of a quiet anterior chamber, the patient was planned for left eye manual small incision cataract surgery (MSICS) and intraocular lens implantation. Intraoperatively, a 2.8-mm sclerocorneal tunnel was made using a crescent blade, and then a side port was made at the 9 o’clock position using a 15-degree angled blade. The anterior chamber was deepened with cohesive viscoelastic (sodium hyaluronate). Dense posterior synechiae restricted pupillary dilation, so a gentle stream of viscoelastic was injected along the posterior iris surface, separating the iris from its adhesions to the lens capsule. This method of controlled viscodissection-viscosynechiolysis helped in releasing the posterior synechiae with relatively no trauma to the iris. 

**Figure 2:**
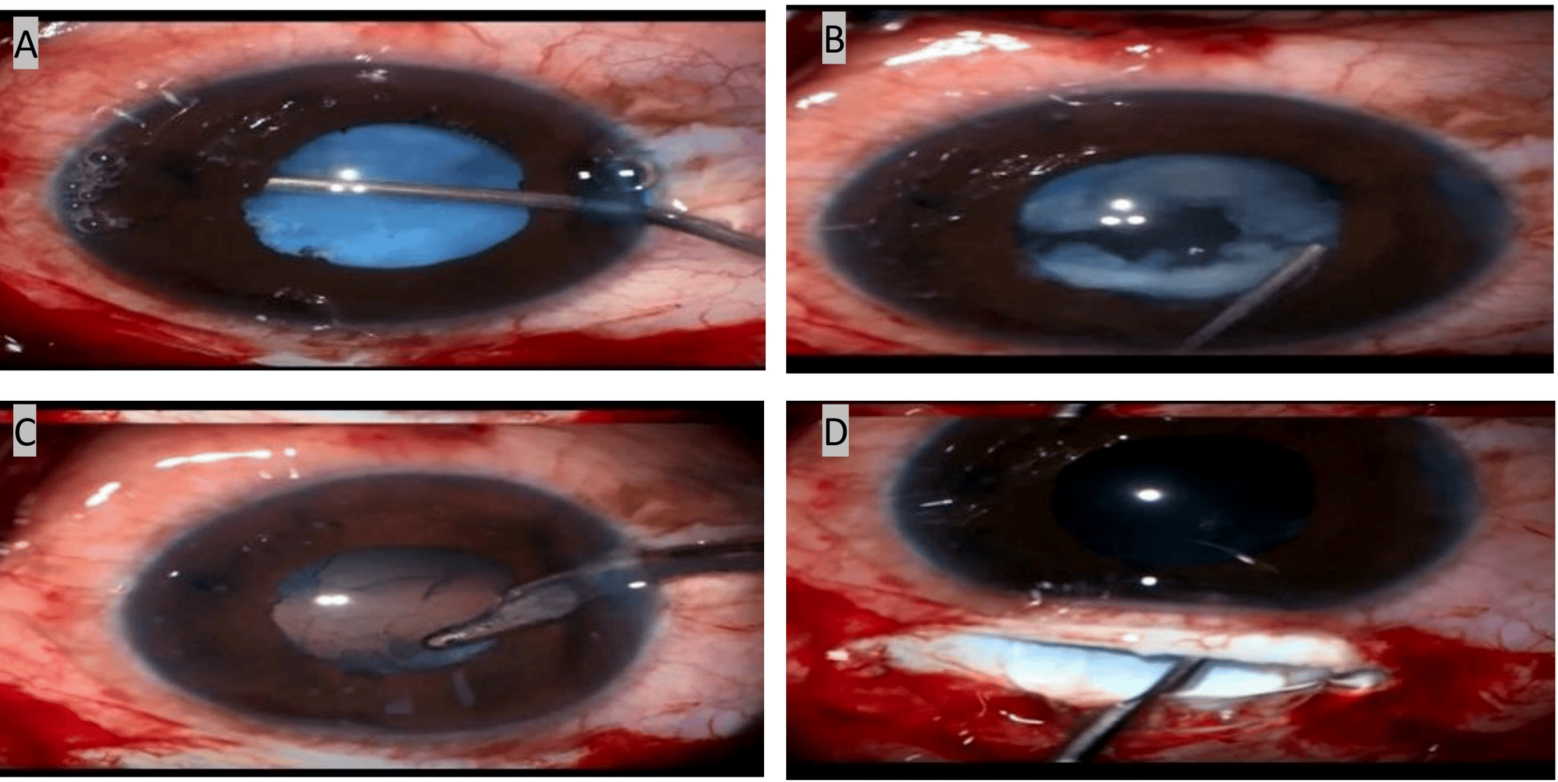
Intraoperative pictures taken through Zeiss Lumera 700* surgical microscope showing (A) viscosynechiolysis, (B) breadcrumb nucleus, (C) cortex aspiration by Simcoe irrigation-aspiration cannula, and (D) IOL implantation *Zeiss, Baden-Württemberg, Germany IOL: intraocular lens

**Figure 3:**
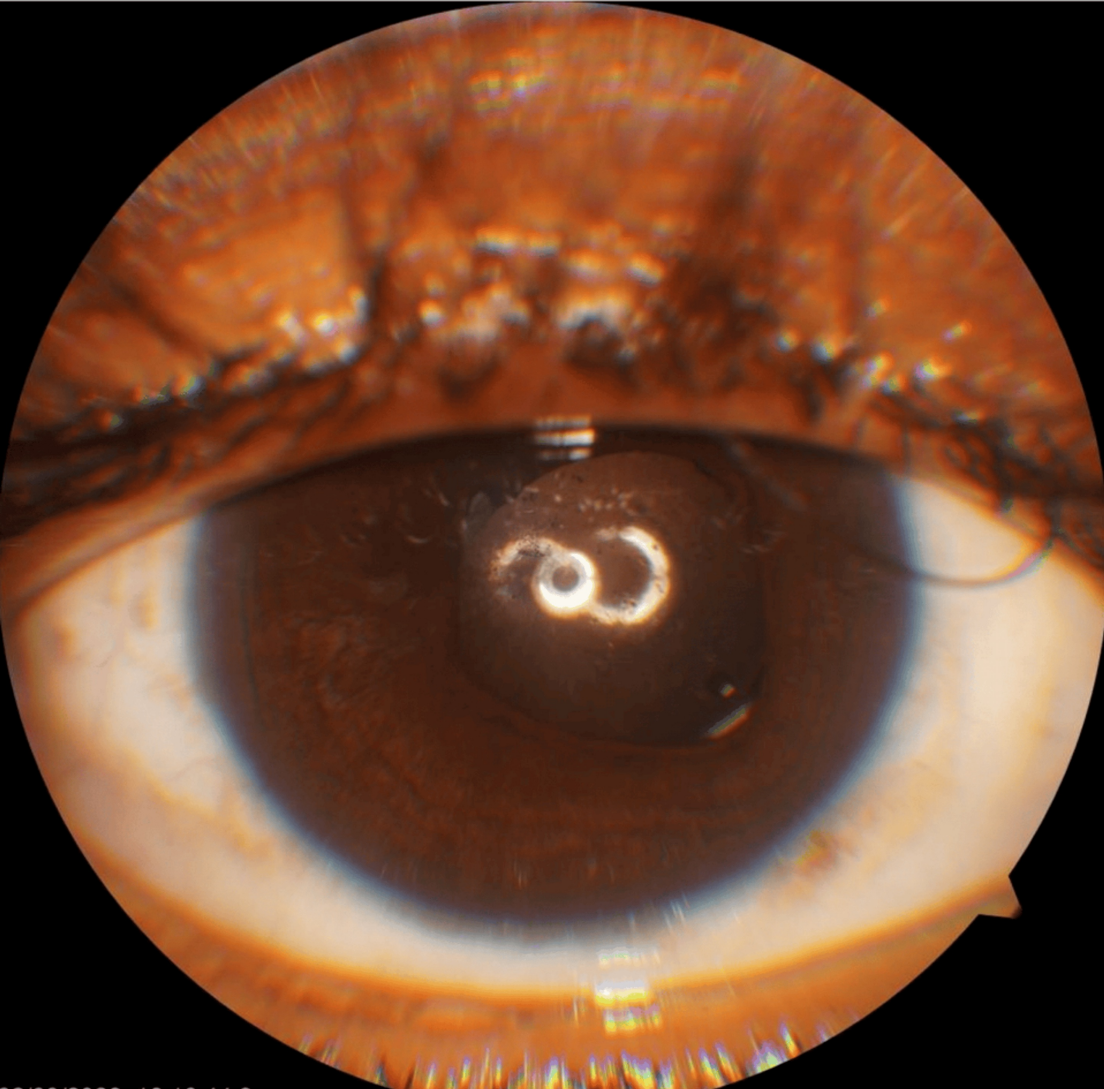
Postoperative image showing implanted intra ocular lens after cataract extraction

Can-opener capsulotomy was then performed using a cystitome. The lens was noted to have a friable consistency resembling breadcrumbs. Controlled manual aspiration was performed using a Simcoe irrigation-aspiration cannula with viscoelastic support, but at the same time, too much of the ocular viscosurgical device was avoided to prevent zonular stress. Cortical aspiration was completed, and a posterior chamber intraocular lens was implanted. The intraoperative course was uneventful, and postoperative recovery was stable with manageable inflammation. Intraoperative pictures are shown in Figure 2. Surgical steps done through the Zeiss Lumera 700 surgical microscope are shown in Video 1. A postoperative image is shown in Figure 3.

**Video 1:** Surgical video showing various operative steps of complicated uveitic cataract

## Discussion

Uveitic cataract surgery differs in quite many ways from age-related cataract surgery due to the presence of chronic intraocular inflammation, formation of posterior synechiae, and altered lens consistency [3]. Of the total eyes undergoing cataract surgery, uveitis eyes comprise approximately 1.2% [4]. Due to repeated attacks of inflammation, fibrotic posterior synechiae between the iris and the anterior lens capsule are formed, which often result in poor pupillary dilation and thereby increase the risk of intraoperative complications [5]. In addition, chronic inflammation can lead to weakness of zonules and alter nuclear consistency, making surgical maneuvers more challenging. These factors necessitate careful surgical planning preoperatively and atraumatic intraoperative techniques.

Posterior synechiae are commonly encountered during management of uveitic cataracts and can significantly limit pupillary dilation. Traditionally, mechanical synechiolysis using instruments like iris hooks was practiced widely, but it may result in iris trauma, bleeding, and even further inflammation [6]. Viscosynechiolysis offers a safer alternative by using viscoelastic to gently separate the iris from the anterior lens capsule through controlled viscodissection, preventing any undue trauma to the iris. This technique allows gradual release of adhesions while minimizing mechanical manipulation of the iris, thereby reducing the risk of intraoperative complications and postoperative inflammation.

Another important intraoperative challenge to keep in mind is the altered consistency of the lens nucleus in inflammatory cataracts. In the current case, the nucleus demonstrated a friable, breadcrumb-like consistency, which warranted an aspiration-based removal rather than conventional nucleus removal strategies. Recognizing such nuclear characteristics is essential because the use of unnecessary surgical maneuvers may increase zonular stress and intraocular inflammation, particularly in eyes with compromised zonules. More manipulation in an already inflamed eye is always going to increase the amount of inflammation in uveitic eyes. Controlled aspiration using a Simcoe irrigation-aspiration cannula enables safe removal of the nucleus while preserving capsular and zonular integrity [7].

The present case also highlights the importance of being flexible in surgical technique based on intraoperative challenges. The combined use of viscosynechiolysis for safe release of posterior synechiae and secondly aspiration-based management of a soft nucleus represents a practical surgical strategy in selected cases of uveitic cataract. Such fine intraoperative decision-making can help minimize surgical trauma, maintain intraocular stability, and reduce postoperative inflammatory response.

However, this report describes a single case, and larger case series are required to validate the reproducibility, safety, and long-term outcomes of this approach. Additionally, optimal postoperative outcomes in uveitic cataract surgery depend not only on surgical technique but also on the postoperative control of ocular inflammation using appropriate topical corticosteroid therapy.

## Conclusions

Viscosynechiolysis is an effective, minimally traumatic method for managing posterior synechiae in uveitic cataract surgery. Recognition of the breadcrumb-like nucleus consistency guides aspiration-based management, reducing zonular stress and phaco energy use. Adapting surgical strategy to lens consistency is essential for optimizing outcomes in complicated cataracts.
